# The Dilemma of Road Safety in the Eastern Province of Saudi Arabia: Consequences and Prevention Strategies

**DOI:** 10.3390/ijerph17010157

**Published:** 2019-12-24

**Authors:** Arshad Jamal, Muhammad Tauhidur Rahman, Hassan M. Al-Ahmadi, Umer Mansoor

**Affiliations:** 1 Department of Civil Engineering, King Fahd University of Petroleum & Minerals, KFUPM Box 655, Dhahran 31261, Saudi Arabia; arshad.jamal@kfupm.edu.sa (A.J.); ahmadi@kfupm.edu.sa (H.M.A.-A.); umerkhan190@gmail.com (U.M.); 2 Department of City and Regional Planning, King Fahd University of Petroleum & Minerals, KFUPM Box 5053, Dhahran 31261, Saudi Arabia

**Keywords:** road safety, traffic crashes, logistic regression, GIS, prevention and mitigation strategies, Saudi Arabia

## Abstract

Road traffic crashes (RTCs) are one of the most critical public health problems worldwide. The WHO Global Status Report on Road Safety suggests that the annual fatality rate (per 100,000 people) due to RTCs in the Kingdom of Saudi Arabia (KSA) has increased from 17.4 to 27.4 over the last decade, which is an alarming situation. This paper presents an overview of RTCs in the Eastern Province, KSA, from 2009 to 2016. Key descriptive statistics for spatial and temporal distribution of crashes are presented. Statistics from the present study suggest that the year 2012 witnessed the highest number of crashes, and that the region Al-Ahsa had a significantly higher proportion of total crashes. It was concluded that the fatality rate for the province was 25.6, and the mean accident to injury ratio was 8:4. These numbers are substantially higher compared to developed countries and the neighboring Gulf states. Spatial distribution of crashes indicated that a large proportion of severe crashes occurred outside the city centers along urban highways. Logistic regression models were developed to predict crash severity. Model estimation analysis revealed that crash severity can be attributed to several significant factors including driver attributes (such as sleep, distraction, overspeeding), crash characteristics (such as sudden deviation from the lane, or collisions with other moving vehicles, road fences, pedestrians, or motorcyclists), and rainy weather conditions. After critical analysis of existing safety and infrastructure situations, various suitable crash prevention and mitigation strategies, for example, traffic enforcement, traffic calming measures, safety education programs, and coordination of key stakeholders, have been proposed.

## 1. Introduction

Road traffic crashes (RTCs) have become a critical public health concern worldwide. The World Health Organization (WHO) and International Transport Forum (ITF) annual reports suggest that approximately 1.35 million people are killed and up to 50 million are injured in RTCs, costing over USD 520 billion globally [1,2]. The current trends reveal that if proper preventive measures are not adopted, then by the year 2030, road traffic injuries are predicted to be the seventh leading cause of death across all age groups, which at present is the leading cause of death for the 15–29 year old age group [3]. The same report also states that 9 out of 10 lives lost to traffic are in low- to middle-income countries, even though these countries share only 48% of the world’s registered vehicles. Economic development has increased the rate of motorization throughout the world, and in particular in low- and middle-income countries [4]. The increased usage and ownership of motor vehicles demands better roads with effective road safety and protection measures. Despite this massive human and economic loss, efforts to combat this global challenge are limited [5,6,7].

Previous studies found that frequencies and severities of RTC occurrence can be contributed to several factors, including drivers’ attributes (age, gender, eyesight, seat belt usage, etc.), vehicle characteristics (vehicle type, vehicle height, vehicle age, tire condition, etc.), roadway characteristics (number of lanes, lane width, shoulder width, pavement surface condition, roadside condition, etc.), crash characteristics (speed at impact, crash type), and environmental conditions (visibility, weather condition, etc.) [8,9,10,11,12,13,14,15,16]. Previous studies have identified numerous factors in order to suggest effective countermeasures. Globally, various preventive measures have been effectively adapted to reduce the burden of RTCs [17,18,19]. Over-speeding, non-compliance to seat belt usage, and use of mobile phones while driving are three primary factors associated with high incidence and severity of RTCs [20]. For example, by devising and enforcing suitable preventive measures, Germany has succeeded in reducing its traffic mortality rate by 69% and severity by more than 50% [21]. Similarly, authorities in New York and several other U.S. cities have been able to reduce the number of fatalities by improving the driving environment, lowering speed limits, and strict enforcement of traffic violations [22,23].

Kingdom of Saudi Arabia (KSA) is the largest country in the Arab states with an area of approximately 2,149,690 km^2^. The country is a member of the “Group of Twenty” (G-20) major world economies. The motorization rate in the KSA has increased rapidly since the oil boom in the early 1970s. Within the last decade, it has increased by almost 67% due to the migration of expatriates from various parts of the world, resulting in rapid urban expansions of major cities [24,25]. These expatriates have diverse backgrounds and attitudes, testing, and training systems for obtaining licenses and driving behaviors. The enormous growth in road infrastructure has resulted in increased road networks with an exponential increase in RTCs. These road crashes are one the major causes of fatalities in the KSA. The WHO Global Status Report on Road Safety reports that the annual fatality rate per 100,000 people due to RTCs in the KSA has increased from 17.4 to 27.4 since the last decade, which is the worst among the countries in the region and is significantly above fatality rate for other G-20 nations such as the USA, the United Kingdom, Japan, and Australia [3]. The economic losses due to RTCs are estimated to be approximately 4.3% of the KSA’s GDP [26]. A study conducted by Turki et al. suggests that more than 19 individuals have lost their lives daily, and approximately 4 people were injured every hour due to RTCs on KSA roads [27]. The majority of victims are young and in the economically more productive age group. According to morbidity and mortality records from the Ministry of Health (MOH), roughly 81% of total fatalities in hospitals are due to RTCs, and nearly 20% of the beds are occupied by RTC victims [28]. Statistics from previous studies for KSA also revealed that the majority of these crashes occurred due to over-speeding and non-compliance with traffic regulations, which can be prevented by strict enforcement [29,30,31,32,33].

Few studies are available in the existing literature that have examined the patterns, trends, and relationships between driving behavior and crash severity in various cities of the KSA. One such study was conducted for 287 young (aged 18–24) drivers [34]. A structural equation modeling approach was adopted to quantify the relationships between driving behaviors and accident involvement. The findings indicated that young drivers can be classified into three distinct categories, these being speedy driving, aggressive driving, and error making. In another similar study, the relationship between different features of crashes, drivers, and vehicles in three different cities of the KSA was investigated [35]. It was concluded that both frequencies and severities of RTCs are significantly higher among young drivers (26–33 years of age) and those driving between 11:00–17:00. The study also revealed that a lower proportion of chauffeurs at fault understood the traffic signs compared to those not at fault. Further, the logit models developed during the research suggested that driver’s behavior, traffic sign recognition, type of vehicle had a statistically significant role in the fault behavior of drivers. In the KSA, road traffic injuries (RTIs) are the third leading cause of death and account for 80% of all trauma admissions in hospitals [36,37]. Recently, the effect of distracted driving on crash severity has been investigated, finding that drivers using a mobile phone while driving have 44% higher chances of being involved in a severe crash [38].

The above studies examined data prior to 2000 and no follow-up studies have been performed to analyze the trends and patterns of vehicle crashes in Saudi cities in general, but particularly in the case of the Eastern Province. This study fulfills that gap by examining the vehicle crash pattern between the years 2009–2016 for the cities in the Eastern Province of the KSA. It also utilized binary logistic regression modeling techniques to predict crash severity within the study area on the basis of the available data. Finally, the study carried out a critical review of the safety and road infrastructure conditions in the study area, proposing suitable counter-measures and mitigation strategies to overcome the burden of RTCs across the region. In the following sections, Section 2 provides a brief description of the study area. Section 3 discusses the data collected and the methods used in the study. The results are presented in Section 4 and are discussed in Section 5. Section 6 is dedicated to the detailed analysis of existing road safety and infrastructure conditions, proposing suitable counter-measures and mitigation strategies. Finally, the concluding remarks and limitations of the study are provided in Section 7.

## 2. Study Area

**Figure 1:**
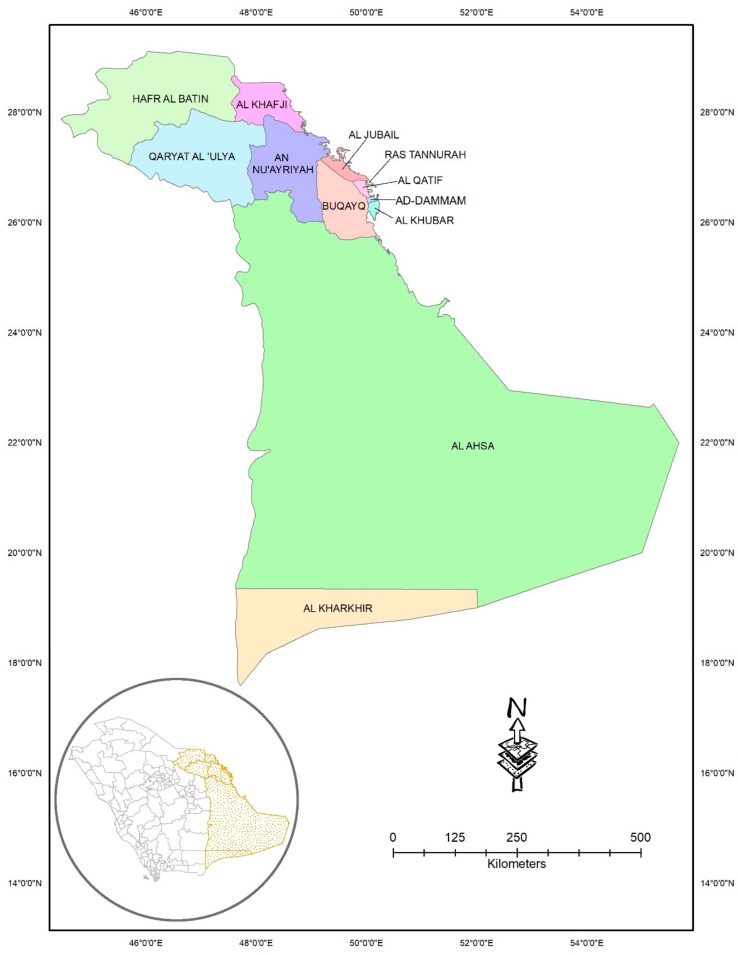
Study area encompassing the Eastern Province along with its 12 cities.

The entire Eastern Province of the KSA was considered as the area of study (Figure 1). The province contains an area of 672,522 km^2^ with a total population of almost 5.1 million [39]. It is reported that the number of registered vehicles in the Eastern Province has increased from 2,971,356 in 2011 to 5,274,251 in 2017 [40]. This rapid increase in motorization across the province has resulted in serious safety concerns. The province is the hub of oil industries in the KSA, which is continuously expanding. The province hosts the Arabian American Oil Company (Saudi ARAMCO), which is the world’s largest oil company. A significant proportion of the population constitutes expatriates coming from diverse backgrounds and cultures, hence generating a heterogeneous road user population.

## 3. Data Collection and Analysis

RTC data for the entire province was acquired from the traffic police department Dammam for the years 2009–2016. A total of 38,780 crashes were reported during this period; however, after cleaning and mining the data, only 30,520 were found valid and used for subsequent analysis. Property damage crashes were excluded from the analysis because of several important missing pieces of information. Descriptive analysis and logistic regression were conducted using the SPSS statistical package (version 25, IBM, Arnmonk, NY, USA), whereas spatial analysis was carried out using the ArcGIS software (ArcGIS 10.6, ESRI, Redlands, CA, USA). A random sample of traffic crashes was selected for crash severity predictive analysis using logistic regression. A similar percentage of fatal and non-fatal crashes was used for analysis in order to avoid bias towards any specific severity class. A fatal crash is defined as any crash resulting in at least one fatality. Driver characteristics, crash type, crash reason, time of the day, and weather conditions were used as input variables. The output class was crash divided into two classes in terms of severity, with these being either fatal or non-fatal.

## 4. Results

### 4.1. Temporal Variations of RTCs

The monthly distribution of the crashes between 2009 and 2016 is shown in Table 1. A total of 30,520 of severe crashes (crashes with fatality or injury) were reported. It can be observed (Table 1) that the highest mean monthly crashes occurred during January (357), followed by December (348), July (333), and August (324). On the contrary, the months of September (297), October (297), May (301), and June (1090) witnessed the lowest number of crashes. The standard deviation for mean monthly crashes observed in October was the largest (88) and was the smallest (44) during May. In terms of year-wise comparison, the total number of crashes was at 3242 at the beginning of 2009 and steadily declined, hitting a low point of 2886 during the year 2010. The total number of crashes then sharply increased in 2011 (3494) and 2012 (4914), and then continued to gradually decrease afterward with 4204 in 2013, 4173 in 2014, 4087 in 2015, and finally 3520 crashes during 2016. Mean annual crashes were slightly less than 4000, with a corresponding standard deviation of 648 crashes per annum. Table 2 summarizes the total number of fatalities and injuries resulting from the RTCs occurring between the periods considered in this study.

It can be observed from Table 2 that a total of 9113 fatalities and 44,298 injuries (both major and minor) occurred during this period. The year 2014 witnessed the highest number of fatalities with a total number of 1252. This was followed by the years 2015 and 2012, where the total fatalities were at 1229 and 1222, respectively. Despite the lower number of crashes reported during 2016 compared to preceding years, the number of fatalities (1214) during the same year were comparable to previous years. Likewise, the highest number of injuries (6674) were observed during 2012, and the lowest (4512) in 2010.

### 4.2. RTC Variations among the Cities of the Eastern Province

**Figure 2:**
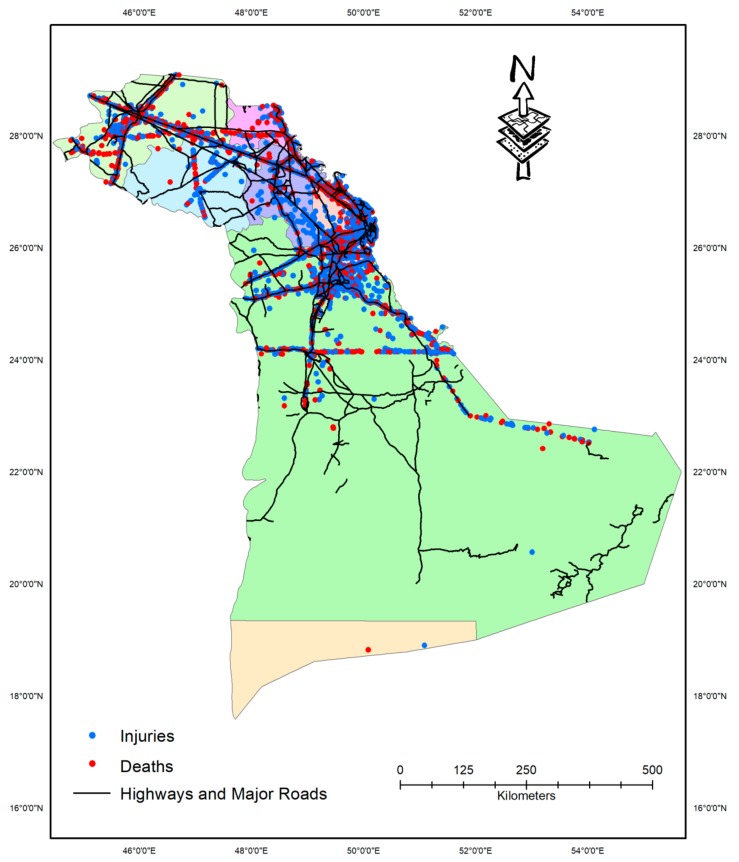
Spatial distribution of crashes among different cities in the Eastern Province.

Table 3 presents the yearly distribution of crashes by different cities in the Eastern Province, whereas Figure 2 shows the spatial distribution of all the injured and fatal crashes in each of the cities. Spatial distributed of crashes indicated that a large proportion of fatal crashes occurred on the outskirts of cities along the highways. It can be observed from Table 3 that Al-Ahsa contributed to the greatest proportion of crashes occurring at different years, whereas the Khafji region had the lowest. Al-Ahsa had the highest mean number of annual crashes (1217) as well, with a correspondingly high standard deviation of 437. Mean annual crashes were 612 in Dammam, 432 in Hafr Al-Batin, 319 in Jubail, and 257 in Dhahran city. The cities of Ras Tanura (80), Khafji (85), Al-Naimiyah (121), and Al-Kobar (237) had the lowest number of annual mean crashes. 

The annual crash fatality rates by different cities are shown in Table 4. The fatality rates given are average annual fatalities per 100,000 population for each region in the respective years. It can be seen that Abqaiq had the highest fatalities rates, exceeding an index value of 100 during most of the years. This value is significantly higher than average fatality rates in different states in the United States, where they are around 10. The mean fatality rates in Al-Naimiyah, Dhahran City, and Khafji were 97.1, 77, and 66.5, respectively. On the other hand, fatality rates for Al-Khobar were the lowest (8.2), followed by Dammam (10.2), Qatif (12.5), and Ras Tanura (14). Fatality rate distribution for different cities across different years did not follow any specific trend.

### 4.3. RTC Distribution by Prevailing Crash Causes and Types

**Figure 3:**
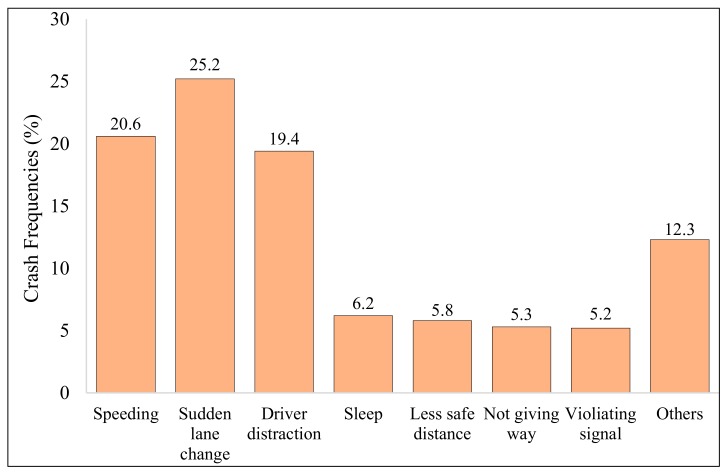
Distribution by prevailing crash causes.

Figure 3 shows the predominant causes of reported crashes, including over-speeding, sudden lane change, driver distraction, sleeping, keeping a short safe distance, not giving way, and non-compliance to traffic signals, among others. Sudden lane changes, over-speeding, and driver distraction accounted for a significant proportion of these crashes with percentages of 25.2%, 20.60%, and 19.40%, respectively. Sleeping while driving contributed to 6.2% of total crashes, whereas crashes occurring due to inadequate safe distance were slightly less than 6%. Similarly, not giving way and violating signals each contributed to around 5% of total crashes. The category “others” consisted of other reasons such as slippery surfaces, violating pedestrian signals, faulty tires, faulty steering wheel, brake failure, engine failure, unprotected work-zone, highway without a fence, and warning signs and signals, among others. Because the proportion of each of these crashes was less than 1.5%, they were not presented individually.

**Figure 4:**
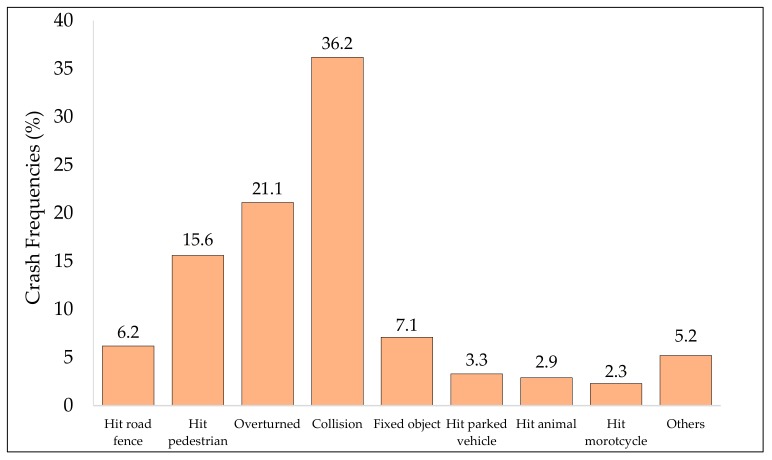
Distribution by prevailing crash types.

**Figure 5:**
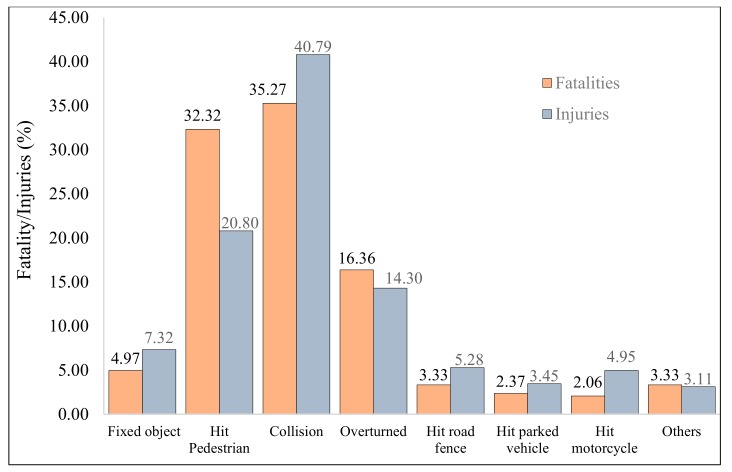
Distribution of fatalities/injuries with crash types.

Figure 4 presents the distribution of crashes by different types such as hitting a road fence; vehicle overturning; hitting a pedestrian and animal collisions (both rear-end and head-on); vehicle overturning; hitting fixed object, parked vehicles, or a motorcyclist, among others. Head-on (32%) and rear-end (68%) crashes were grouped into the single category “collisions” to avoid any congestion in Figure 4 and Figure 5. It is evident (from Figure 4) that collision was the most predominant crash type reported, contributing to almost one-third of the total crashes. The second and third major crash categories were crashes due to vehicle overturning (21.1%) and hitting pedestrians (15.6%). Hitting fixed objects and road fences each constituted approximately 7% of the total crash types, with colliding with parked vehicles and hitting an animal being slightly lower at around 3%. Hitting a tree, traffic sign, waste container, or bicycle/motorcycle, as well as falling from a bridge, were each less than 1%.

Figure 5 provides information on the percentage of fatalities/injuries resulting from different crash types. It can be observed from the plot that collisions (hitting another vehicle) were responsible for a significant proportion of fatalities and injuries reported. Vehicle head-on collisions resulted in approximately one-third (33%) of the total fatalities and around two-fifths (40%) of the total number of injuries. Vehicle overturning is another primary crash type resulting in over 20% of fatalities and approximately 33% of the total injuries. This is followed by crash types due to vehicle overturning, where the proportion of fatalities was estimated at around 17%, whereas that of injuries was at about 14%. Fatalities and injuries from hitting fixed objects and road fences ranged between 3% and 8%. A relatively lower number of fatalities (2.06%) and injuries (4.95%) resulted from hitting pedestrians. Similarly, fatalities and injuries around 3% each emerged from crash types listed in another category column described previously.

### 4.4. Crash Severity Modeling Using Logistic Regression

Logistic regression approach was adopted for crash severity modeling. Logistic regression methods were preferred because conventional regression analysis is generally used when the output is continuous. Logistic regression is appropriate if the output is categorical, having binary classes. Logistic regression is very efficient and easy to interpret. This technique is more informative compared to other classification techniques as it provides a relationship between output and all its input features. This technique not only gives the measure of relevance of a feature but also gives its direction of the association, whether positive or negative. Logistic regression methods have been successfully used for reasonably accurate crash severity modeling in previous studies [41,42,43]. Crash severity was defined in two classes, these being whether a crash was fatal or non-fatal, given a list of explanatory variables at a selected confidence level. The main aim of logistic regression, just like any other statistical technique, is to find the best fit. However, it differs from linear regression in terms of the response variable, which is binary in nature for logistic regression. Crash severity, which is the dependent variable in this study, has a binary nature. The two classes for the dependent variable are a fatal crash and a non-fatal crash. The simple mathematical expressions adopted for logistic regression modeling can found in previous studies [44,45], and shown below:

(1) $$p\left(fatal\;crash\right)=\;\pi \left(x\right)=\frac{e^{g\left(x\right)}}{1+e^{g\left(x\right)}}$$

where *g*(*x*) represents the functional form of input variables, and is given by

(2) $$g\left(x\right)=\;\beta_{0}+\beta_{1}x_{1}+\beta_{2}x_{2}+\cdots +\beta_{n}x_{n}.$$

Subsequently, the probability of non-fatal crash can be estimated using the equation:

(3) $$p\left(non-fatal\;carsh\right)=1-p\left(fatal\;carsh\right)=\;1-\pi \left(x\right),$$

(4) $$p\left(non-fatal\;crash\right)=\frac{1}{1+e^{g\left(x\right)}}.$$

Logistic regression uses the maximum likelihood algorithm in order to determine the coefficients, which make the observed outcome most probable [46]. A random sample of 2500 traffic crash data points was selected for crash severity predictive analysis using logistic regression. A similar percentage of fatal and non-fatal crashes was used for analysis in order to avoid bias towards any specific severity class. In our study, backward stepwise regression approach using SPSS software was followed to model the independent variables from our dataset. Although stepwise may have some limitations, it nonetheless has obvious advantages over conventional methods of analysis. For example, stepwise logistic regression has the capacity to manage a lot of potential predictor variables, adjusting the model to pick the best predictor variables from the accessible choices. It is computationally faster compared to automatic model selection techniques. It gives better insight into the predictor variables just by looking at the way they are removed or added. All the categorical independent variables were changed to dummy variables, as shown in Table 5. In backward regression process, insignificant variables were successively eliminated. Significant variables with *p*-values less than 0.05 in last step are shown in Table 6.

The estimated results of the logistic model using a backward regression approach are shown in Table 6. The magnitude and sign of coefficients explain the effect of a factor on the risk of fatality. A positive sign of the coefficient (β) shows a higher probability of fatality related to the corresponding factor, whereas the negative sign indicates a lesser likelihood of fatality.

Model estimation results from logistic regression analysis are shown in Table 6. The variables shown and included in Table 6 were highly significant at a 95% confidence level. Logistic regression analysis revealed that crash severity can be attributed to different explanatory variables, including key driver attributes, crash characteristics, and weather conditions. Regarding driver attributes, it was found that the probability of a fatal crash increased for drivers who fell asleep while driving. This is obvious, as the vehicle would be out of control if the driver was sleeping. This was evident from the positive sign of the estimated coefficient for sleep (β = 1.023; *p* = 0.01). The higher value of the odds ratio (Exp β = 2.781) also shows that the chances of a fatality were high. Likewise, the coefficient for speeding was β = 0.676 with a *p*-value of 0.001, and magnitude of the odds ratio of Exp β = 1.966 was also high. It is evident from the current knowledge and intuition that speeding is the primary cause of fatal crashes. Although many other factors have a confounding effect on speeding, some of these factors include geometric design features of the roadway, traveling terrain, and visibility conditions, among other factors. By controlling these factors, the effect of speeding can be mitigated. Our analysis reveals that speeding increases the likelihood of fatality.

Similarly, there were a number of crash characteristics that had a strong bearing on whether a crash would be a fatal or a non-fatal one. Significant factors in this category included sudden lane deviation or hitting another moving vehicle, a road fence, an electric pole, a pedestrian, or a motorcyclist. Lower *p*-values and higher odd ratios for parameters listed in this category indicated their significance, as shown in Table 6. The signs for all coefficients were positive and well intuitive because there was a high probability of being involved in a fatal crash for each of the crash scenarios mentioned. Also, the odds ratio for all of the variables was more than 1.0, revealing that the chances of a fatality were high. The highest chances of fatality among these variables were for hitting a pedestrian barrier, which had a coefficient value β of 1.865, followed by hit pedestrian crashes, having a β value of 1.289. It is interesting to note from Table 6 that although the variable of violating red signal was significant, it had a negative coefficient (β = −1.572), indicating that the chance of fatality was low for such instances, which seems counter-intuitive compared to previous studies [47,48,49,50]. This anomaly could be attributed to the fact that red-light cameras accompany the majority of signalized intersections in Saudi Arabia. As evident from previous studies, the presence of red-light cameras results in fewer head-on crashes because the cameras prevent red-light running [51,52]. Drivers tend to brake abruptly in the dilemma zone due to the presence of red-light cameras. Drivers’ conflicting decisions in the dilemma zone result in rear-end crashes [53,54]. The rear end crashes result in slight injury or property damage, obvious from many studies [55,56,57,58]. A previous study conducted for the nearby city of Riyadh also indicated that on average non-intersection crashes were more fatal compared to intersection crashes [59]. Similarly, the probability of low crash fatality on a rainy day having a large negative value for coefficient (β = −2.532) also appears conflicting to previous studies [60,61,62]. However, it can be argued from several previous studies that inclement weather may cause a significant reduction in vehicle speed [63,64,65]. During rainy weather, drivers tend to drive slowly, so even if a crash occurs, the chances of a fatality are lower. Weather in Saudi Arabia is usually clear throughout the year, and it rains very rarely. Hence, a large proportion of drivers are not familiar/accustomed to rainy driving conditions, meaning that they become more cautious by reducing their speeds during rainy weather, and thus the probability of high crash severity is low. The coefficient for constant was β = −5.938 with a *p*-value of 0, and the magnitude of the odds ratio was Exp β = 0.003. The coefficient for constant incorporated the effect of all other factors on the likelihood of fatality.

## 5. Discussion

This paper presents an overview of road traffic crashes in the Eastern Province, KSA, between 2009 and 2016. A total of 30,520 crashes were witnessed during this period, resulting in 9113 total fatalities. The mean monthly distribution of crashes indicated that months from May to November carried the lowest number of crashes, with a slight increase during the months of July and August. This trend can be attributed partially due to prevailing warm temperatures during most parts of the year, and partly because of the majority of expatriates leave the province for vacations during summer. Moreover, locals also prefer to travel abroad or to move to nearby states in order to avoid the scorching heat during the hot summer. This mean monthly distribution of crash trends is somewhat similar to the neighboring Gulf states of Oman [66] and Bahrain [67], having similar demographics and driving environments. There are four peak traffic periods that are prone to a greater number of crashes occurring during the daytime. These peak periods for the Eastern Province are similar to a study conducted for the kingdom of Bahrain [67], a state having a similar driving culture and is merely 30 km away from Dammam, the capital of the Eastern Province. Comparing the annual crash trends during 2012 and 2013, the highest number of crashes were recorded, which was followed by a steady declining trend. This reduction in annual crashes was mostly due to the implementation of traffic enforcement and the imposition of heavy traffic fines in the following year. Al-Ahsa and Dammam cities contribute to almost 52% of total crashes. This is because of the fact that the mentioned cities have a major proportion of the population and thus the corresponding number of registered vehicles in the Eastern Province.

Over-speeding, sudden lane changes and the driver distraction were the primary causes that accounted for about 65% of total crashes in the region. The proportion of different causes reported herein is comparable to a study previously conducted for the Dammam metropolitan [68]. However, these statistics for proportions of different causes leading to road traffic crashes are different from previous research studies conducted for other cities in the KSA [26,69]. The most predominant crash types were vehicle collisions (36.3%), vehicle overturning (21.1%), and hitting pedestrians (15.6%), which formed almost three-quarters of total crashes reported. These numbers are somehow different from a study carried out in neighboring Oman, where collisions, vehicle overturning, and hitting pedestrians were reported to be 58%, 4.8%, and 3.0%, respectively [66].

The mean annual accident-to-injury ratio for the whole Eastern Province was 8:4, which is slightly different from 8:6, a ratio reported for one such previous study, as well as the international ratio of 8:1 [26]. Similarly, the mean annual fatality rate (per 100,000 people) for the study area was 25.6, which is near to the overall fatality ratio of 27.4 for the entire KSA, and higher than 17.4, a value presently prevailing for the globe [3]. Mean fatality rate for the Eastern Province, and the entire KSA is ranked third among twenty Eastern Mediterranean countries, marginally above Libya and Iran in the region [70]. Likewise, the fatality rate for the Eastern Province is substantially higher than neighboring Gulf states [66,71,72], India [73], China [74], the United States, the United Kingdom, Canada, Australia [75], and other Asian countries [76]. This is an alarming situation for a G-20 high-income country. Model estimates of logistic regression models indicate that several significant factors would determine the level of severity of a crash. Some of these key factors include driver attributes (such as sleep, distractions, speeding), crash characteristics (collisions with moving vehicles, road fences, pedestrians, and motorcyclists), and rainy weather conditions. These findings are in close agreement with a couple of recent studies conducted for other Saudi cities [59,77].

## 6. Mitigation and Prevention Strategies

Fatalities and injuries due to RTCs can be minimized and prevented by adopting proper road safety strategies. A wide range of road safety enhancement strategies have been proposed by previous studies to tackle this global challenge [7,78,79,80,81,82]. Addressing the worsening traffic safety situations in the KSA, efforts were initiated in 2003 with the aim of improving safety on national roads by identification of proper remedial measures and proposition of strategies for future implementation. Steps taken to reduce the burden of RTCs in KSA include enforcement of seatbelt and traffic regulations, installation of speed cameras throughout the KSA, and emergency medical treatment units, among others. Recently, a traffic control program called SAHER was implemented to reduce traffic crashes and optimize the efficiency of the traffic system. SAHER is an automated system adopted for controlling traffic using a digital network of cameras connected to the central information center. However, the implementation of these actions seems inadequate, as the traffic safety situations have only been marginally improved. There is a major gap between law enforcement and policy, and actions toward effective implementation of these strategies are under serious question; thus, the situation needs to be re-examined. Detailed analysis of the study results (from Figure 4 and Table 6) indicated that a large proportion of total crashes and crash severity occurring in the study area were associated with distracted driving (20%), speeding (about 20%), and fatigue driving (approximately 6%). The following passages provide key strategies for mitigating and preventing RTCs that need to be proactively implemented throughout the Eastern Province to improve the deteriorating road safety situations. Listed counter-measures and strategies have been suggested after comprehensive data analysis in the study area, careful review of the local crash database, and multiple site visits to selected crash-prone locations to improve the road safety situation in the KSA in general and in the Eastern Province in particular.

### 6.1. Advanced Crash Data Recording System

Accurate and organized crash data reports are fundamental to conduct a thorough analysis, suggest improvements and establish priorities, and to develop strategies for mitigating and preventing RTCs [83]. Several studies in the past have indicated the significance of the crash database for providing suitable counter-measures [84,85,86]. The current methods for data collection, compiling, and recording needs comprehensive improvements. Although crash reports are prepared at crash locations, they are non-analytical and rudimentary for any purpose beyond general reporting and aggregation, which makes the data less useful for suggesting tangible safety improvements. Therefore, it is recommended that a comprehensive crash data recording system be adopted containing information on driver characteristics (age, gender, physical condition), characteristics of vehicles involved (type, age, tire conditions), roadway characteristics, precise location and time of the crash, weather conditions, and collisions diagrams. The current dataset does not allow for detailed statistical analysis to establish correlations between different risk factors such as young drivers, driving under the influence of alcohol, driving during the night, driver fatigue, driver distraction (mostly due to using of mobile phones), and several other road- and vehicle-related risk factors. The present crash record also lacks precise coordinates data, which is vital in identifying high-risk locations/road segments. Standard road safety data collection and analysis mechanisms are also poor, showing a lack of seriousness toward saving human lives, valuable property, and infrastructure, as well as improving overall safety situations on national roads.

### 6.2. Legislation and Enforcement

Legislation and strict enforcement can significantly help in improving road safety situations [76,87,88,89]. Presently, legislation regarding different driving laws, including seatbelt law, helmet use for motorcyclists, and speed limit law, exists across all major cities. However, legislation on child restraint law, on-street parking, and compliance with some traffic control regulations needs to be enforced immediately. A low level of enforcement abrogates the efforts made through legislation to enhance road safety. The government has increased fines for violating red signals and the use of mobile phones while driving, which is encouraging in terms of restoring and maintaining road safety. The most critical challenge is to enforce the speed limit among young drivers aged 21 and younger, who are found to be the most frequent violators [37].

### 6.3. Safety Education Programs and Awareness Campaigns

Safety education programs and awareness campaigns are less frequent and below standards, despite the prevailing critical road safety in the KSA. These programs and campaigns have been found to be extremely vital in enhancing road safety situations worldwide [90,91,92]. Thus, the regional traffic departments in the KSA should provide low-cost safety education programs focusing on traffic safety issues such as compliance with national driving laws, drunk driving, speeding, mobile phone use, and any other type of distraction. Similarly, awareness campaigns for road safety in public should be initiated through seminars, TV, radio, traffic police, and pamphlet distribution. Safety awareness campaigns can be designed to target a specific group, for example, the young drivers in the KSA context that are mostly involved in road crashes. Safety education programs and awareness campaigns, together with strict enforcement of laws, are believed to increase road safety considerably.

### 6.4. Induction of Traffic and Speed Calming Measures

Traffic calming measures are designed to improve safety for motorists, cyclists, and pedestrians. Traffic calming measures including speed bumps, speed humps, raised crosswalks, and safety islands, among other forms, helping to control speed and regulate traffic. Erecting these various measures has been found to be quite useful in promoting road safety [93,94,95,96]. During the analysis for the current study, it was observed that the majority of crashes in the Eastern Province occurred near intersections with improper speed calming measures. It is also recommended that channelization at intersections is provided that can reduce the number of conflict points without significantly reducing the approach speeds. Similarly, lack of appropriate access control on freeways and other major highways can negatively affect the traffic operations, as randomly located entry and exit points lead to an abrupt reduction in speed, impedance to traffic flow, and inefficient use of traffic capacity. Providing suitable traffic calming measures on the entrance and exit ramps can be beneficial in raising the safety levels.

### 6.5. Emergency Medical Care Units for Victims

The first hour after a crash has occurred is commonly referred to as “golden hour” [97,98], during which if the victim receives proper first aid, the chances of survival and reduction in severities of other injuries are high. Unfortunately, current system for emergency medical care of crash victims in KSA is lagging international standards. An emergency medical care unit solely dedicated to crash victims should be built, as it was mentioned previously that more than four injuries happen every hour on national roads [27]. Small scale medical care units should be located on busy roads for preliminary treatment. It is recommended that response time for ambulances should be minimized, and trained health personnel should accompany the crash victims in shifting and transporting to nearby healthcare units. It has been observed that the most common cause of fatalities post-crash is oxygen breakdown. Thus, the first aid crew arriving first at the scene should be provided with oxygen. One recent study conducted in Tabuk, KSA, indicated that pre-hospital care for crash victims could be enhanced using mobile phone smart technologies by proper coordination with nearby available facilities/services [99].

### 6.6. Rigorous Identification and Treatment of Crash Hotspots

Crash hotspots may be defined as road segments and locations that have an unusually high concentration of crashes than expected [100]. Current crash data lack precise coordinates for the crash location, which makes it difficult to identify crash hotspots. Several previous studies have suggested that accurate identification of crash hotspots is the first and key step towards effective highway safety management process through the adoption of appropriate counter-measures [101,102,103,104,105]. Error in the process may lead to inefficient use of resources and thus hindering the national goals of achieving the desired road safety targets. For the KSA scenario, it is recommended that traffic police personnel and crew who first arrive at the crash scene should be equipped with high-quality global positioning system (GPS) devices to record the exact coordinates of such spots. This location data can be used later on for the analysis of crash hotspots. The common feature of these crash hotspots is either that these locations are geometrically deficient or that drivers make mistakes at these sites due to information overload. After the crash hotspots are identified, priorities are established and are treated with suitable counter-measures.

### 6.7. Engagement and Coordination of Key Stakeholders

Effective engagement and coordination of concerned stakeholders are vital factors for mitigating crash severity. At present, there is low-level coordination between the traffic police department and emergency treatment units. It is recommended that this coordination between different parties, that is, police, ambulance services, emergency healthcare units, and hospitals, be made efficient. This will ensure minimal possible time for shifting and transporting crash victims, and thus several precious lives may be saved, whereas for others, injury severity may be mitigated.

## 7. Conclusions

This study presents an overview of road traffic crashes in the Eastern Province, KSA, from 2009 to 2016. A total of 30,520 crashes were reported that resulted in a total of 9113 fatalities and 44,298 injuries (both major and minor) during this period. Descriptive statistics and spatial distribution pertaining to the crashes were presented. The results suggest that between 2012 and 2014, the number of crashes was the highest. When analyzing monthly crash patterns, January, July, and December generally had the highest number of crashes, whereas September, October, and June witnessed a low frequency of crashes. Distribution by cities showed that Al-Ahsa had the highest proportion of crashes, whereas Khafji had the lowest in the study period. It was also found that the fatality index for the entire Eastern Province was 25.4, whereas the mean annual crash to injury ratio was 8:4. The present study revealed that some of the predominant causes of crashes include over-speeding, sudden lane change, driver distraction, sleeping, and keeping an inadequate safe distance. The logistic regression analysis results showed that sleeping; distractions; speeding; collisions with moving vehicles, road fences, pedestrians, and motorcyclists; and rainy weather conditions were some of the significant factors contributing to crash severity. To mitigate the huge socio-economic burden of traffic crashes suffered annually, different potential crash prevention strategies, for instance, traffic enforcement, traffic calming measures, safety education programs, and coordination of key stakeholders, have been proposed.

This study had several limitations that should be acknowledged. First, data utilized in the study had minimal demographic and socio-economic characteristics of the drivers, which is an important aspect. The geographic coordinates were also not available for all the crash locations, and, therefore, they were not useable for any geospatial analysis in the study. Second, detailed information pertaining to property damage was not available in the dataset. Finally, the number of deaths recorded in the database was only for victims who passed away at the crash scenes. It is possible that victims were taken to hospital and died later. However, their deaths were not recorded in the accident database with the Dammam Traffic Department. Future studies should focus on resolving some of these limitations by utilizing further detailed data to detect other possible patterns of vehicle crashes in the province. Studies should also focus on using advanced geospatial (i.e., hotspot analysis) and artificial intelligence techniques, including deep machine learning, game theory, and artificial neural network (ANN), among other techniques, for modeling the severity of crashes. Finally, they should also examine crash patterns for other provincial cities.

## Figures and Tables

**Table 1:** Temporal distribution of crashes during the study period (2009–2016).

Year	January	February	March	April	May	June	July	August	September	October	November	December	Total Crashes
2009	402 (12.4)	238 (7.34)	262 (8.08)	277 (8.54)	266 (8.2)	260 (8.02)	389 (12)	235 (7.25)	228 (7.03)	204 (6.29)	213 (6.58)	268 (8.27)	3242 (100)
2010	297 (10.29)	256 (8.87)	222 (7.69)	225 (7.8)	246 (8.52)	225 (7.8)	249 (8.63)	251 (8.7)	187 (6.48)	183 (6.34)	217 (7.52)	328 (11.36)	2886 (100)
2011	288 (8.24)	292 (8.36)	277 (7.93)	276 (7.9)	265 (7.58)	260 (7.44)	251 (7.18)	385 (11.02)	326 (9.34)	313 (8.96)	294 (8.41)	267 (7.64)	3494 (100)
2012	349 (7.1)	410 (8.34)	432 (8.79)	367 (7.47)	332 (6.76)	383 (7.79)	480 (9.77)	471 (9.58)	460 (9.36)	474 (9.65)	356 (7.25)	400 (8.14)	4914 (100)
2013	350 (8.33)	295 (7.02)	331 (7.87)	313 (7.45)	358 (8.52)	354 (8.42)	375 (8.92)	341 (8.1)	333 (7.92)	314 (7.47)	381 (9.06)	459 (10.92)	4204 (100)
2014	414 (9.92)	363 (8.7)	371 (8.89)	365 (8.75)	349 (8.36)	332 (7.96)	341 (8.17)	326 (7.81)	297 (7.12)	294 (7.05)	354 (8.48)	367 (8.79)	4173 (100)
2015	363 (8.88)	298 (7.29)	403 (9.86)	318 (7.78)	324 (7.93)	309 (7.56)	331 (8.1)	329 (8.05)	303 (7.41)	308 (7.54)	376 (9.2)	425 (10.4)	4087 (100)
2016	391 (11.11)	316 (8.97)	330 (9.38)	342 (9.72)	268 (7.61)	309 (8.78)	248 (7.05)	253 (7.18)	243 (6.9)	283 (8.04)	265 (7.53)	272(7.73)	3520 (100)
Total	2854	2468	2628	2483	2408	2432	2664	2591	2377	2373	2456	2786	30,520
Mean	357	309	329	310	301	304	333	324	297	297	307	348	3815
SD	46	56	72	49	44	53	82	79	83	88	69	76	648

Note: values in parenthesis indicate percentages.

**Table 2:** Frequency of year-wise fatalities and injuries during the study period (2009–2016).

Year	Fatalities	Injuries
2009	1145	5029
2010	995	4512
2011	1058	5267
2012	1222	6674
2013	998	5665
2014	1252	6082
2015	1229	5822
2016	1214	5247
Total	9113	44,298
Mean	1139	5537
SD	107	671

**Table 3:** Distribution of crashes by cities between 2009–2016.

Year	Abqaiq	Al-Ahsa	Al-Khobar	Al-Naimiyah	Dammam	Dhahran	Hafr Al-Batin	Jubail	Khafji	Qatif	Ras Tanura	Total Crashes
2009	115 (3.55)	712 (21.96)	139 (4.29)	117 (3.61)	505 (15.58)	194 (5.98)	522 (16.1)	298 (9.19)	102 (3.15)	443 (13.66)	95 (2.93)	3242 (100)
2010	97 (3.36)	714 (24.74)	234 (8.11)	81 (2.81)	411 (14.24)	57 (1.98)	504 (17.46)	284 (9.84)	93 (3.22)	335 (11.61)	76 (2.63)	2886 (100)
2011	259 (7.41)	922 (26.39)	193 (5.52)	91 (2.6)	649 (18.57)	114 (3.26)	522 (14.94)	358 (10.25)	90 (2.58)	204 (5.85)	92 (2.63)	3494 (100)
2012	188 (3.83)	1550 (31.54)	368 (7.49)	137 (2.79)	807 (16.42)	429 (8.73)	437 (8.89)	529 (10.77)	95 (1.93)	236 (4.8)	138 (2.81)	4914 (100)
2013	149 (3.54)	1031 (24.52)	327 (7.78)	158 (3.76)	836 (19.89)	368 (8.75)	433 (10.3)	364 (8.66)	94 (2.24)	400 (9.51)	44 (1.05)	4204 (100)
2014	144 (3.45)	1806 (43.28)	231 (5.54)	116 (2.78)	534 (12.8)	345 (8.27)	342 (8.2)	295 (7.07)	62(1.49)	266 (6.36)	32 (0.76)	4173 (100)
2015	202 (4.94)	1708 (41.79)	209 (5.11)	134 (3.28)	564 (13.8)	262 (6.41)	352 (8.61)	230 (5.63)	91 (2.23)	251 (6.14)	84(2.06)	4087 (100)
2016	123 (3.49)	1293 (36.73)	191 (5.43)	131 (3.72)	586 (16.65)	288 (8.18)	343 (9.74)	196 (5.57)	53 (1.51)	234 (6.65)	82 (2.33)	3520 (100)
Total	1277	9736	1892	965	4892	2057	3455	2554	680	2369	643	30,520
Mean	160	1217	237	121	612	257	432	319	85	296	80	3815
SD	54	437	75	25	147	128	79	102	18	87	32	648

Note: values in parenthesis indicate percentages.

**Table 4:** Crash fatality rates by cities (fatalities/100,000 people).

Year	Abqaiq	Al-Ahsa	Al-Khobar	Al-Naimiyah	Dammam	Dhahran	Hafr Al-Batin	Jubail	Khafji	Qatif	Ras Tanura
2009	146.2	30	8.1	147.1	12.3	87.1	43.8	35.8	81.3	18.4	21.1
2010	135.2	26.2	10.4	84.9	7.7	29.7	49.9	31.4	64.9	15.1	9.8
2011	125.5	28.9	9.6	78.2	11.5	33.8	41.6	42.5	64.6	12.7	9.5
2012	87.7	31.5	9.3	112.3	12.8	146.3	40.6	38	74.1	7.9	20
2013	119	24.3	7.3	75.9	7.4	91.2	34.2	30.2	86.9	12.2	20.9
2014	108.3	37.7	5.9	88.3	11.4	72.9	43.2	33.3	54.7	11.9	10.1
2015	155.7	27.7	7.8	90.2	10.1	81.9	49.2	29.9	59.1	10.5	12.7
2016	117.5	27.5	7	100	8.6	73	43.9	24.6	46.7	11.5	8.2
Mean	124.4	29.2	8.2	97.1	10.2	77	43.3	33.2	66.5	12.5	14

**Table 5:** Explanatory variables used in the model.

Variable Name	Variable Type	Variable Description
**Driver Related Factors**		
Sleep	Categorical	1 = Sleep; otherwise = 0
Distractions	Categorical	1 = Distractions; otherwise = 0
Speeding	Categorical	1 = Speeding; otherwise = 0
Exhaustion	Categorical	1 = Exhaustion; otherwise = 0
Alcohol use	Categorical	1 = Alcohol use; otherwise = 0
**Accident Type**		
Hit a moving vehicle	Categorical	1 = Hit a moving vehicle; otherwise = 0
Hit an animal	Categorical	1 = Hit an animal; Otherwise = 0
Hit a road fence	Categorical	1 = Hit a road fence; otherwise = 0
Hit an electric pole	Categorical	1 = Hit an electric pole; otherwise = 0
Hit a side barrier	Categorical	1 = Hit a side barrier; otherwise = 0
Hit a parked vehicle	Categorical	1 = Hit a parked vehicle; otherwise = 0
Hit a motorcycle	Categorical	1 = Hit a motorcycle; otherwise = 0
Hit a pedestrian	Categorical	1 = Runover; otherwise = 0
Hit a tree	Categorical	1 = Hit a tree; otherwise = 0
**Accident Reason**		
Violate red signal	Categorical	1 = Override the red signal; otherwise = 0
Sudden lane deviation	Categorical	1 = Sudden lane deviation; otherwise = 0
Violate stop sign	Categorical	1 = Violate stop sign; otherwise = 0
Not giving way	Categorical	1 = Do not give priority; otherwise = 0
Vehicle drift	Categorical	1 = Drift; otherwise = 0
Not enough space	Categorical	1 = Not enough space; otherwise = 0
Violation of pedestrian signal	Categorical	1 = Violation of pedestrian signal; otherwise = 0
Slipping	Categorical	1 = slipping; otherwise = 0
Absence of warning signs	Categorical	1 = Lack of warning signs; otherwise = 0
No signal	Categorical	1 = No signal; otherwise = 0
Faulty tires	Categorical	1 = Faulty tires; otherwise = 0
Faulty steering wheel	Categorical	1 = Faulty tires; otherwise = 0
Engine combustion	Categorical	1 = Faulty tires; otherwise = 0
Overloading	Categorical	1 = Overloading; otherwise = 0
**Lighting Condition**		
Day	Categorical	1 = Day; otherwise = 0
Night	Categorical	1 = Night; otherwise = 0
**Weather**		
Clear	Categorical	1 = Clear; otherwise = 0
Cloudy	Categorical	1 = Cloudy; otherwise = 0
Rainy	Categorical	1 = Rainy; otherwise = 0
Dusty	Categorical	1 = Dust; otherwise = 0

**Table 6:** Model’s parameter estimates for significant variables.

Explanatory Variable	Coefficient β	Standard Error.	Wald Statistic	Significance * p-Value	Odds Ratio
**Driver Characteristics**					
Sleep	1.023	0.42	5.982	0.01	2.781
Distractions	0.327	0.105	9.683	0.002	1.387
Speeding	0.676	0.09	62.724	0.001	1.966
**Crash Type**					
Hit a moving vehicle	0.564	0.06	93.724	0.02	1.757
Hit a road fence	0.723	0.16	21.548	0.03	2.06
Hit an electric pole	0.784	0.23	11.227	0.01	2.19
Hit a pedestrian	1.865	0.26	50.675	0.02	6.457
Hit a motorcycle	1.289	0.67	3.694	0.004	3.628
**Crash Reason**					
Violate red signal	−1.572	0.381	16.985	0.02	0.208
Sudden lane deviation	0.242	0.085	8.084	0.004	1.273
**Weather**					
Rainy	−2.532	1.112	5.182	0.003	0.079
Constant	−5.938	0.48	155.86	0	0.003

Note: * parameters significant at α = 0.05; -2log-likelihood ratio= 4481.32; chi-square = 165.07; *p*-value < 0.001.
